# Supplementary material for: FAT-switch-based quantitative S-nitrosoproteomics reveals a key role of GSNOR1 in regulating ER functions
Source: Nat Commun. 2023 Jun 5;14:3268. doi: 10.1038/s41467-023-39078-0 (PMC10241878; doi:10.1038/s41467-023-39078-0)
Supplement: Supplementary file 1 — Supplementary Information [file 41467_2023_39078_MOESM1_ESM.pdf]

FAT-switch-based quantitative S-nitrosoproteomics reveal a key role of GSNOR1 in regulating ER functions

Qin et al.

Supplementary Information

This file includes:

Supplementary Figure 1 to Figure 5 with figure legends, Supplementary Table I and II with references, and list of Supplementary Datasets.

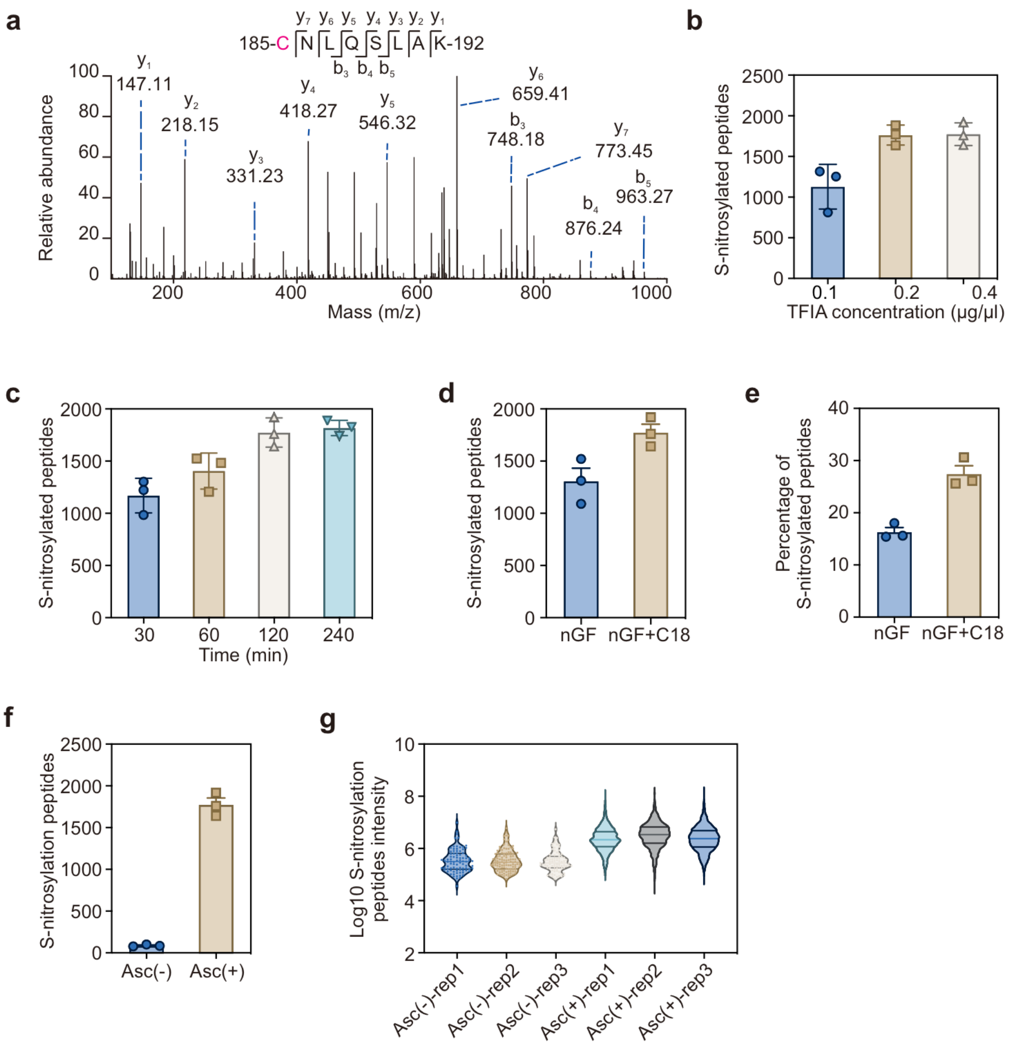

Supplementary Fig. 1 FAT-switch method shows higher sensitivity than biotin-switch method. a MS/MS spectrum of C^{185}NLQSLAK peptide from PYL4 protein. The increase of 417.04 Da on the cysteine residue indicates that the site was labeled by TFIA. **b** The number of S-nitrosylated peptides identified by LC-MS/MS in different TFIA concentration. Error bars indicate SD (n = 3 biologically independent samples). **c** The number of S-nitrosylated peptides identified by LC-MS/MS in different incubation time with TFIA. Error bars indicate SD (n = 3). **d** The number of identified S-nitrosylated peptides with or without C18 enrichment Error bars indicate SD (n = 3 biologically independent samples). **e** The percentage of identified S-nitrosylated peptides in all peptides with or without C18 enrichment. Error bars indicate SD (n = 3). **f** The number of S-nitrosylation peptides identified by FAT-switch method with or without ascorbate (Asc), three replicates were performed. Error bars indicate SD (n = 3 biologically independent samples). **g** Boxplots of $\log_{10}$ MS/MS intensity of all identified S-nitrosylation peptides in fluorous tag (F-tag) switch approach with or without ascorbate (Asc). The horizontal lines in the boxplots indicate the median; whisker caps and circles indicate the 10th-90th and 1st-99th percentiles, respectively.

a

TMT	Material	Peptides(ug)	Bio. rep.
126	WT	200	1
127	*hot5-4*	200	
128	WT	200	2
129	*hot5-4*	200	
130	WT	200	3
131	*hot5-4*	200	

b

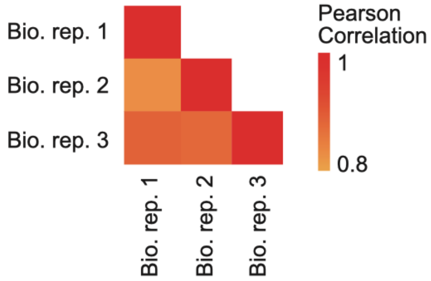

c

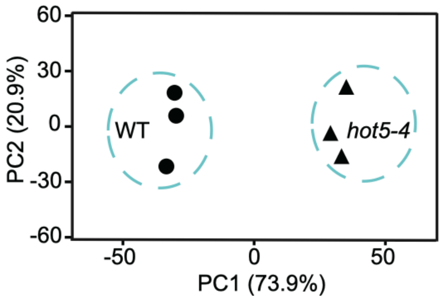

Supplementary Fig. 2 Comparison of the biological replicates in TMT-sixplex labeling.
a TMT six-plex labeling of wild type and *hot5-4* seedlings. **b** Pearson's correlation of *hot5-4*/WT S-nitrosylation peptide rations between biological replicates. **c** Principal component analysis (PCA) of the S-nitrosylation sites across all six samples.

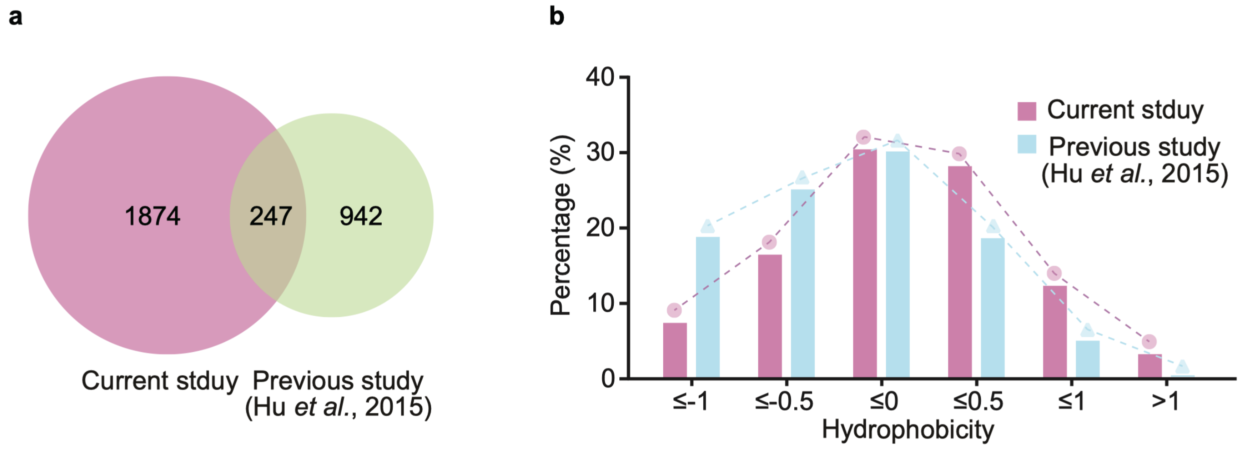

Supplementary Fig. 3 Comparison of S-nitrosylated peptides identified by this study and previous studies. a The number of S-nitrosylated peptides identified in this study and previous study. **b** Hydrophobic distribution of all identified S-nitrosylation peptides in in this study (red) and previous study (blue).

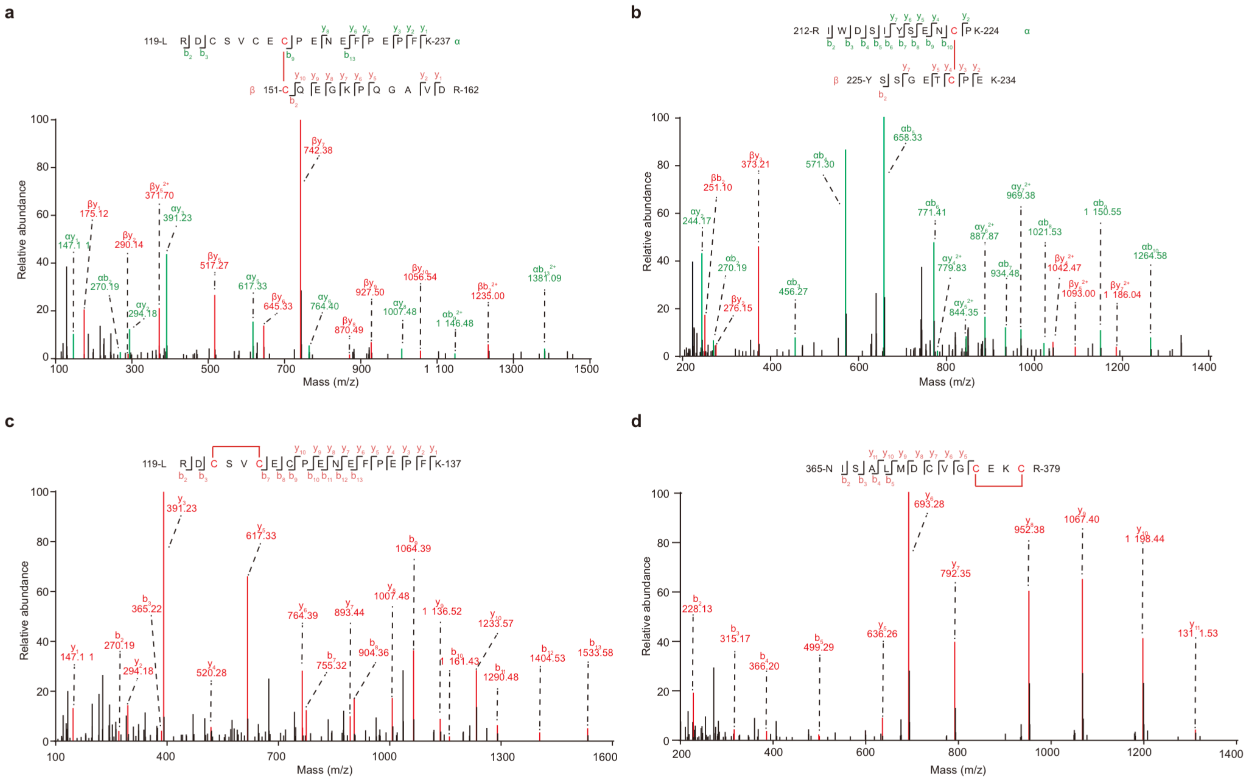

e

Disulfide bonds	Mock (abundance)			+GSNO (abundance)			Fold change	p-value
	Rep.1	Rep.2	Rep.3	Rep.1	Rep.2	Rep.3	(+GSNO/mock)	(two-tail unpaired *t* test)
Cys122/125-Cys125/127	2057605	1424758	1124161	1793137	1698657	1461621	1.075	0.7125
Cys122/125/127-Cys151	51765791	21773156	33913334	41139908	23190721	62101841	1.177	0.6795
Cys222-Cys231	4446837278	5149300235	4111001612	2636146205	2122100534	1607926496	0.464	0.0046
Cys375-Cys378	57353871	54151836	22437319	10713737	7762897	9450333	0.208	0.0341
Cys71-Cys337	4313569	3556208	3803589	882772	1049407	993729	0.251	0.0002
138-KPFVPGLPSDDLK-150	86217381913	101977389709	88878636414	104790323795	85289964593	77647564856	0.966	0.7578

Supplementary Fig. 4 Identified disulfide bonds with in ERO1 by LC-MS/MS. **a** MS/MS spectrum of the disulfide bonds linked peptides between Cys122/125/127 and Cys151 in ERO1 protein**. b** MS/MS spectrum of the disulfide bonds linked peptides between Cys222 and Cys231 in ERO1 protein**. c** MS/MS spectrum of the disulfide bonds linked peptides between Cys122-Cys125 in ERO1 protein**. d** MS/MS spectrum of the disulfide bonds linked peptides between Cys375 and Cys378 in ERO1 protein**. e** the relative abundance of the disulfide bonds linked peptide in ERO1 protein after incubating with or without GSNO.

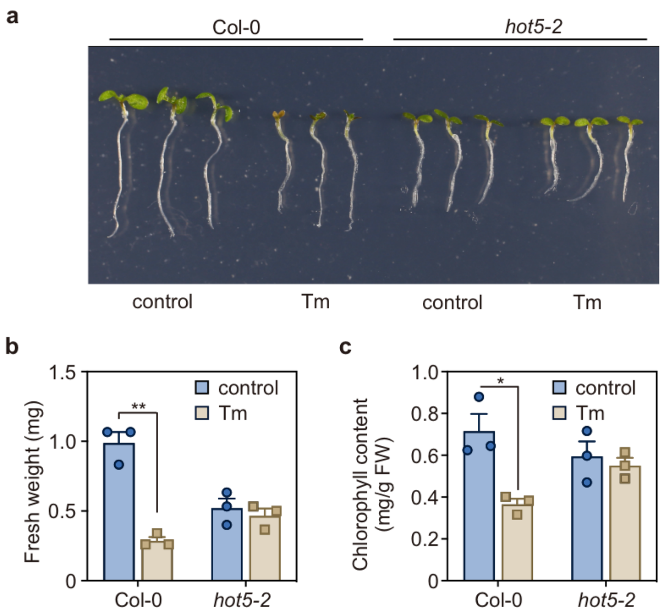

Supplementary Fig. 5. *hot5-2* mutant shows the tolerance phenotype of ER stress under Tm treatment. **a** Phenotype of wild type (Col-0) and *hot5-2* with or without Tm treatment. Photograph were taken after growing at 22 ºC for 9 days. **b** Fresh weight of Col-0 wild type and *hot5-2* plants with or without Tm treatment. Error bars, SD (each dot represents one biological replicate with 3 seedlings). ***p* < 0.01, two-tail unpaired *t*-test. **c** Chlorophyll content of WT and *hot5-4* plants with or without Tm treatment. Error bars, SD (each dot represents one biological replicate with 3 seedlings). **p* < 0.05, two-tailed unpaired *t*-test.

Supplementary Table I. Comparison of SNO proteomics studies in plants.

Method	Material	Number of SNO proteins/peptides/sites	Amount of starting material/condition/mutant	Quantify-cation	Year	Ref.
Biotin-switch	Arabidopsis	105 proteins		No	2005	1
Biotin-switch	Arabidopsis	16 proteins	hypersensitive response	No	2008	2
Biotin-switch	*Brassica juncea*	20 proteins	cold	No	2009	3
Biotin-switch	*Citrus aurantium*	49 proteins	salinity	No	2009	4
Biotin-switch	Arabidopsis leave mitochondria	11 proteins	harpin	No	2010	5
Biotin-switch	Arabidopsis cells	53 peptides	2 mg proteins	No	2011	6
Biotin-switch	Arabidopsis	58 proteins	*Pst* DC3000/*gnsor1*	No	2011	7
Biotin-switch	Pea peroxisomes	6 proteins		No	2012	8
Biotin-switch	tobacco	11 peptides	*Phytophthora cryptogea*	No	2012	9
Biotin-switch	Arabidopsis	5 proteins	auxin	No	2013	10
Biotin-switch	pea	29 proteins	salinity	No	2013	11
Biotin-switch	Potato leaves	80 proteins		No	2013	12
Biotin-switch	*Brassica juncea*	110 spots	cold	No	2013	13
Biotin-switch	Arabidopsis plantlets	62 peptides	cold stress	No	2014	14
Biotin-switch	*Brassica juncea*	271 proteins	PAs	No	2013	15
Biotin-switch	*Brassica juncea*	48 proteins	cold stress	No	2014	16
Biotin-switch	Populus	32 proteins	Acute ozone fumigation	No	2014	17
Biotin-switch	Arabidopsis seedlings	926 proteins, 1,195 peptides	*gsnor1-3*	No	2015	18
Biotin-switch	wheat	44 proteins	dehydration	No	2016	19
Biotin-switch	sunflower	61 proteins	30 µg proteins	No	2018	20
iodoTMT	tomato	334 proteins, 425 sites	1 mg/*GSNOR* RNAi line	Yes	2019	21
Biotin-switch	Cucumber	165 proteins	GSNO/cPTIO/*gsnor1*	No	2019	22
iodoTMT	Tea leaves	191 proteins, 228 sites	1 mg proteins	Yes	2019	23
Biotin-switch	*Lotus japonicus*	281 proteins		No	2020	24
iodoTMT/ iTRAQ	Arabidopsis leave peels	35 proteins, 41 peptides	100 peels/Flg22	Yes	2020	25
FAT-switch	Arabidopsis seedlings	1,595 proteins, 2,121 peptides	0.5 g seedlings/*hot5-4*	Yes	2022	This study

Supplementary Table II. Primers used in this study.

Primers	Sequence
bip3-L	CACGGTTCCAGCGTATTTCAAT
bip3-R	ATAAGCTATGGCAGCACCCGTT
shd-L	GAAGGAAGCATTCAAGGAGCTA
shd-R	TCTTTGATGATAGGGTGTCGTG
bip1,2-L	TCACTTGGGAGGTGAGGACTTT
bip1,2-R	CTCACATTCCCTTCGGAGCTTA
actin-L	GGTAACATTGTGCTCAGTGGTGG
actin-R	AACGACCTTAATCTTCATGCTGC
His-MSB-BamHI-ERO1-F	GAAAACCTCTACTTCCAAGGATCCATGAATTCCAATGTCGGTTTC
His-MSB-NotI-ERO1-R	TCGACTTAAGCATTATGCGGCCGCTTACCAGAATGAGACAGC
His-MSB-BamHI-PDI9-F	GAAAACCTCTACTTCCAAGGATCCATGTATAAATCACCATTA
His-MSB-NotI-PDI9-R	TCGACTTAAGCATTATGCGGCCGCTCACAACTCATCCTTAGA
MSB-ERO1-C337S-F	ACAGCTTCTCCTGTTCCGTTTGATGAA
MSB-ERO1-C337S-R	AACAGGAGAAGCTGTTTGGAGTTTCGG
HOT5-2 GABI_315D11-LP	ATGGTTCGACGCATATTTTTC
HOT5-2 GABI_315D12-RP	GGAAAGAGACCTTCAGGATCC
ero1-3-Salk_096805-LP	GATTCTTGGTTTCGACCCATC
ero1-3-Salk_096805-RP	CTTTAGCGACTTTGGTTGTCG
LBb1.3	ATTTTGCCGATTTCGGAAC
GABI	ATATTGACCATCATACTCATTGC

Reference

1. Lindermayr C, Saalbach G, Durner J. Proteomic identification of S-nitrosylated proteins in Arabidopsis. *Plant Physiol.* **137**, 921-930 (2005).
2. Romero-Puertas MC*, et al.* Proteomic analysis of S-nitrosylated proteins in *Arabidopsis thaliana* undergoing hypersensitive response. *Proteomics* **8**, 1459-1469 (2008).
3. Abat JK, Deswal R. Differential modulation of S-nitrosoproteome of *Brassica juncea* by low temperature: change in S-nitrosylation of Rubisco is responsible for the inactivation of its carboxylase activity. *Proteomics* **9**, 4368-4380 (2009).
4. Tanou G*, et al.* Proteomics reveals the overlapping roles of hydrogen peroxide and nitric oxide in the acclimation of citrus plants to salinity. *Plant J.* **60**, 795-804 (2009).
5. Palmieri MC, Lindermayr C, Bauwe H, Steinhauser C, Durner J. Regulation of plant glycine decarboxylase by s-nitrosylation and glutathionylation. *Plant Physiol.* **152**, 1514-1528 (2010).
6. Fares A, Rossignol M, Peltier J-BÆ. Proteomics investigation of endogenous S-nitrosylation in Arabidopsis. *Biochem. Biophys. Res. Commun.* **416**, 331-336 (2011).
7. Holzmeister C, Fröhlich A, Sarioglu H, Bauer N, Durner J, Lindermayr C. Proteomic analysis of defense response of wildtype Arabidopsis thaliana and plants with impaired NO-homeostasis. *Proteomics* **11**, 1664-1683 (2011).
8. Ortega-Galisteo AP, Rodríguez-Serrano M, Pazmiño DM, Gupta DK, Sandalio LM, Romero-Puertas MC. S-Nitrosylated proteins in pea (*Pisum sativum* L.) leaf peroxisomes: changes under abiotic stress. *J. Exp. Bot.* **63**, 2089-2103 (2012).
9. Astier J*, et al.* Nitric oxide inhibits the ATPase activity of the chaperone-like AAA^+ ATPase CDC48, a target for S-nitrosylation in cryptogein signalling in tobacco cells. *Biochem. J.* **447**, 249-260 (2012).
10. Correa-Aragunde N, Foresi N, Delledonne M, Lamattina L. Auxin induces redox regulation of ascorbate peroxidase 1 activity by S-nitrosylation/denitrosylation balance resulting in changes of root growth pattern in Arabidopsis. *J. Exp. Bot.* **64**, 3339-3349 (2013).
11. Camejo D*, et al.* Salinity-induced changes in S-nitrosylation of pea mitochondrial proteins. *J. Proteomics* **79**, 87-99 (2013).

12. Kato H, Takemoto D, Kawakita K. Proteomic analysis of S-nitrosylated proteins in potato plant. *Physiol. Plant.* **148**, 371-386 (2013).
13. Sehrawat A, Abat J, Deswal R. RuBisCO depletion improved proteome coverage of cold responsive S-nitrosylated targets in *Brassica juncea*. *Front. Plant Sci.* **4**, (2013).
14. Puyaubert J, Fares A, Rézé N, Peltier JB, Baudouin E. Identification of endogenously S-nitrosylated proteins in Arabidopsis plantlets: effect of cold stress on cysteine nitrosylation level. *Plant Sci.* **215-216**, 150-156 (2014).
15. Tanou G*, et al.* Polyamines reprogram oxidative and nitrosative status and the proteome of citrus plants exposed to salinity stress. *Plant Cell Environ.* **37**, 864-885 (2014).
16. Sehrawat A, Deswal R. S-nitrosylation analysis in *Brassica juncea* apoplast highlights the importance of nitric oxide in cold-stress signaling. *J. Proteome Res.* **13**, 2599-2619 (2014).
17. Vanzo E*, et al.* S-nitroso-proteome in poplar leaves in response to acute ozone stress. *PLoS One* **9**, e106886 (2014).
18. Hu J*, et al.* Site-specific nitrosoproteomic identification of endogenously S-nitrosylated proteins in Arabidopsis. *Plant Physiol*. **167**, 1731-1746 (2015).
19. Gietler M, Nykiel M, Orzechowski S, Fettke J, Zagdańska B. Proteomic analysis of S-nitrosylated and S-glutathionylated proteins in wheat seedlings with different dehydration tolerances. *Plant Physiol. Biochem.* **108**, 507-518 (2016).
20. Jain P, von Toerne C, Lindermayr C, Bhatla SC. S-nitrosylation/denitrosylation as a regulatory mechanism of salt stress sensing in sunflower seedlings. *Physiol. Plant.* **162**, 49-72 (2018).
21. Gong B, Shi Q. Identifying S-nitrosylated proteins and unraveling S-nitrosoglutathione reductase-modulated sodic alkaline stress tolerance in *Solanum lycopersicum* L. *Plant Physiol. Biochem.* **142**, 84-93 (2019).
22. Niu L*, et al.* Proteomic investigation of S-nitrosylated proteins during NO-induced adventitious rooting of cucumber. *Int. J. Mol. Sci.* **20**, 5363 (2019).
23. Qiu C*, et al.* First nitrosoproteomic profiling deciphers the cysteine S-nitrosylation involved in multiple metabolic pathways of tea leaves. *Sci. Rep.* **9**, 17525 (2019).
24. Matamoros MA*, et al.* Altered plant and nodule development and protein S-nitrosylation in *Lotus japonicus* mutants deficient in S-nitrosoglutathione reductases. *Plant Cell Physiol.* **61**, 105-117 (2019).
25. Lawrence SR, 2nd, Gaitens M, Guan Q, Dufresne C, Chen S. S-nitroso-proteome revealed in stomatal guard cell response to Flg22. *Int. J. Mol. Sci.* **21**, 1688 (2020).

List of Supplementary Datasets:

Supplementary Dataset 1: S-nitrosylation peptides identified by biotin-switch method

Supplementary Dataset 2: S-nitrosylation peptides identified by FAT-switch method

Supplementary Dataset 3: Quantitative S-nitroso-proteomics by FAT-switch method

Supplementary Dataset 4: S-nitrosylation peptides identified by previous studies

Supplementary Dataset 5: S-nitrosylation peptides enriched in *hot5-4* mutant

Supplementary Dataset 6: Proteomics analysis of WT and *hot5-4* mutant seedlings

Supplementary Dataset 7: GO and KEGG analyses of *hot5-4* enriched S-nitrosylated proteins
