# Supplementary material for: FAT-switch-based quantitative S-nitrosoproteomics reveals a key role of GSNOR1 in regulating ER functions
Source: Nat Commun. 2023 Jun 5;14:3268. doi: 10.1038/s41467-023-39078-0 (PMC10241878; doi:10.1038/s41467-023-39078-0)
Supplement: Supplementary file 3 — Description of Additional Supplementary Files [file 41467_2023_39078_MOESM3_ESM.pdf]

Description of Additional Supplementary Files:

Supplementary Dataset 1: S-nitrosylation peptides identified by biotin-switch method

Supplementary Dataset 2: S-nitrosylation peptides identified by FAT-switch method

Supplementary Dataset 3: Quantitative S-nitroso-proteomics by FAT-switch method

Supplementary Dataset 4: S-nitrosylation peptides identified by previous studies

Supplementary Dataset 5: S-nitrosylation peptides enriched in hot5-4 mutant

Supplementary Dataset 6: Proteomics analysis of WT and hot5-4 mutant seedlings

Supplementary Dataset 7: GO and KEGG analyses of hot5-4 enriched S-nitrosylated proteins
