# Supplementary material for: FAT-switch-based quantitative S-nitrosoproteomics reveals a key role of GSNOR1 in regulating ER functions
Source: Nat Commun. 2023 Jun 5;14:3268. doi: 10.1038/s41467-023-39078-0 (PMC10241878; doi:10.1038/s41467-023-39078-0)
Supplement: Supplementary file 11 — Reporting Summary [file 41467_2023_39078_MOESM11_ESM.pdf]

nature portfolio

Corresponding author(s): Pengcheng Wang

Last updated by author(s): May 15, 2023

Reporting Summary

Nature Portfolio wishes to improve the reproducibility of the work that we publish. This form provides structure for consistency and transparency in reporting. For further information on Nature Portfolio policies, see our Editorial Policies and the Editorial Policy Checklist.

Statistics

For all statistical analyses, confirm that the following items are present in the figure legend, table legend, main text, or Methods section.

n/a	Confirmed	
☐	☒	The exact sample size (*n*) for each experimental group/condition, given as a discrete number and unit of measurement
☐	☒	A statement on whether measurements were taken from distinct samples or whether the same sample was measured repeatedly
☐	☒	The statistical test(s) used AND whether they are one- or two-sided<br>*Only common tests should be described solely by name; describe more complex techniques in the Methods section.*
☐	☒	A description of all covariates tested
☒	☐	A description of any assumptions or corrections, such as tests of normality and adjustment for multiple comparisons
☐	☒	A full description of the statistical parameters including central tendency (e.g. means) or other basic estimates (e.g. regression coefficient) AND variation (e.g. standard deviation) or associated estimates of uncertainty (e.g. confidence intervals)
☐	☒	For null hypothesis testing, the test statistic (e.g. *F*, *t*, *r*) with confidence intervals, effect sizes, degrees of freedom and *P* value noted<br>*Give P values as exact values whenever suitable.*
☒	☐	For Bayesian analysis, information on the choice of priors and Markov chain Monte Carlo settings
☒	☐	For hierarchical and complex designs, identification of the appropriate level for tests and full reporting of outcomes
☒	☐	Estimates of effect sizes (e.g. Cohen's *d*, Pearson's *r*), indicating how they were calculated

Our web collection on statistics for biologists contains articles on many of the points above.

Software and code

Policy information about availability of computer code

Data collection	Raw MS data was collected with Thermo Scientific Xcalibur 3.0.
Data analysis	Identification and quantification of peptides and proteins were analyzed by Proteome Discoverer 2.2.<br>Protein Property Analysis Software 1.1 was used to calculate the hydrophobicity values for SNO peptides.<br>Disulfide bonds in AtERO1 protein were identified by P-link software 2.3.

For manuscripts utilizing custom algorithms or software that are central to the research but not yet described in published literature, software must be made available to editors and reviewers. We strongly encourage code deposition in a community repository (e.g. GitHub). See the Nature Portfolio guidelines for submitting code & software for further information.

Data

Policy information about availability of data

All manuscripts must include a data availability statement. This statement should provide the following information, where applicable:

- Accession codes, unique identifiers, or web links for publicly available datasets
- A description of any restrictions on data availability
- For clinical datasets or third party data, please ensure that the statement adheres to our policy

The mass spectrometry proteomics data have been deposited to the ProteomeXchange Consortium via the PRIDE partner repository with the dataset identifier PXD037504. Source data underlying Figures 1b–d; 2a-f; 3c-g; 4e; 5a-h, as well as Supplementary Figures 1b-g; 2b; 2c; 3a; 3b; 4e; 5a-c are provided as a Source Data

file. Any other data supporting the findings of this study are available within the manuscript and its supplementary files or are available from the corresponding author upon request.

Human research participants

Policy information about studies involving human research participants and Sex and Gender in Research.

Reporting on sex and gender	No Human research participants/Sex/Gender used in this research.
Population characteristics	No Human research participants/Sex/Gender used in this research.
Recruitment	No Human research participants/Sex/Gender used in this research.
Ethics oversight	No Human research participants/Sex/Gender used in this research.

Note that full information on the approval of the study protocol must also be provided in the manuscript.

Field-specific reporting

Please select the one below that is the best fit for your research. If you are not sure, read the appropriate sections before making your selection.

- [x] Life sciences
- [ ] Behavioural & social sciences
- [ ] Ecological, evolutionary & environmental sciences

For a reference copy of the document with all sections, see nature.com/documents/nr-reporting-summary-flat.pdf

Life sciences study design

All studies must disclose on these points even when the disclosure is negative.

Sample size	No statistical methods were used to predetermine sample size. The determined sample size was adequate as the differences between experimental groups was sigificant and reproducible.
Data exclusions	No data were excluded from the analyses.
Replication	The recordings and parameters were reproducible across multiple days and batches. All attempts o replication were successful.
Randomization	Randomization of samples were performed. Seedlings from different plates were collected.
Blinding	The investigators were not blinded to allocation during experiments and outcome assessment. In order to get as objective results as possible, in multiple experiments we had other researchs repeating the experiments.

Reporting for specific materials, systems and methods

We require information from authors about some types of materials, experimental systems and methods used in many studies. Here, indicate whether each material, system or method listed is relevant to your study. If you are not sure if a list item applies to your research, read the appropriate section before selecting a response.

Materials & experimental systems

n/a	Involved in the study	
[ ]	[x]	Antibodies
[x]	[ ]	Eukaryotic cell lines
[x]	[ ]	Palaeontology and archaeology
[x]	[ ]	Animals and other organisms
[x]	[ ]	Clinical data
[x]	[ ]	Dual use research of concern

Methods

n/a	Involved in the study	
[x]	[ ]	ChIP-seq
[x]	[ ]	Flow cytometry
[x]	[ ]	MRI-based neuroimaging

Antibodies

Antibodies used	anti-RPN12a (Agrisera, AS194268), anti-SDIRIP1 (Agrisera, AS132729), anti-PSB33 (Agrisera, AS121852), anti-CNX (Agrisera, AS122365), anti-CRT from Jianming Li's Lab, anti-biotin antibody (Cell Signaling Technology, 7075), anti-his antibody (Cell Signaling Technology, 12698)

Validation

All antibodies used here have been validated by manufacturers or donator.
anti-RPN12a: https://www.agrisera.com/en/artiklar/rpn12a.html
anti-SDIRIP1: https://www.agrisera.com/en/artiklar/raf2-rubisco-accumulation-factor-2.html
anti-PSB33: https://www.agrisera.com/en/artiklar/tef5-rieske-2fe-2s-domain-containing-protein-.html
anti-CNX: https://www.agrisera.com/en/artiklar/cnx1_2-calnexin-homolog-1_2.html
anti-CRT: https://doi.org/10.1016/j.molcel.2007.05.015
anti-biotin antibody: https://www.cellsignal.com/products/secondary-antibodies/anti-biotin-hrp-linked-antibody/7075
anti-his antibody: https://www.cellsignal.com/products/primary-antibodies/his-tag-d3i1o-xp-rabbit-mab/12698
